# Autocrine Activity of Extracellular Vesicles Induced by Icariin and Its Effectiveness in Glucocorticoid-Induced Injury of Bone Microvascular Endothelial Cells

**DOI:** 10.3390/cells11121921

**Published:** 2022-06-14

**Authors:** Qingyu Zhang, Tengqi Li, Zirong Li, Jike Lu, Xinjie Wu, Fuqiang Gao, Wei Sun

**Affiliations:** 1 Department of Orthopedics, Shandong Provincial Hospital Affiliated to Shandong First Medical University, Jinan 250000, China; zhangqingyu@sdfmu.edu.cn; 2 Department of Orthopedics, Graduate School of Peking Union Medical College, China-Japan Friendship Institute of Clinical Medicine, Beijing 100000, China; lizirong0297@163.com; 3 Department of Orthopedics, Peking University China-Japan Friendship School of Clinical Medicine, Beijing 100000, China; litengqi@bjmu.edu.cn (T.L.); wuxinjie@pku.edu.cn (X.W.); 4 Department of Orthopedics, Peking University Shougang Hospital, Beijing 100000, China; 5 Centre for Osteonecrosis and Joint-preserving & Reconstruction, Department of Orthopedics, China-Japan Friendship Hospital, Beijing 100000, China; 6 Department of Orthopedics, Beijing United Family Hospital (BJU), Beijing 100000, China; jike.lu@ufh.com.cn; 7 School of Medical Sciences, Faculty of Medicine, The University of New South Wales, Sydney, NSW 2052, Australia; 8 Department of Molecular Medicine and Surgery, Karolinska Institutet, 17177 Stockholm, Sweden

**Keywords:** bone microvascular endothelial cells, glucocorticoid, icariin, protein array, extracellular vesicles

## Abstract

Glucocorticoids could induce injury and apoptosis of bone microvascular endothelial cells (BMECs) in the femoral head, which is associated with the development of osteonecrosis and osteoporosis. Icariin is a prenylated flavonol glycoside isolated from Epimedium brevicornum, serving as the main active pharmaceutical constituent to treat bone loss. Currently, the impact of the autocrine activity of extracellular vesicles (EVs) induced by icariin on the glucocorticoid-induced injury of BMECs is still to be confirmed. In this study, EVs were isolated from BMECs treated with and without icariin by super-speed centrifugation. Although icariin treatment would not significantly change the size and total protein content of BMECs-derived EVs, expression of EVs-carried vascular endothelial growth factor (VEGF) and transforming growth factor β1 (TGF-β1) was enhanced and numerous miRNAs involved in cell proliferation and apoptosis were upregulated (e.g., hsa-miR-1469 and hsa-miR-133a-5p) or downregulated (e.g., hsa-miR-10b-5p) (*p* < 0.05). A total of 29 differentially expressed inflammatory factors were detected between the EVs secreted by BMECs from the Icariin-treated group and the Model group. The EVs secreted by BMECs could improve cell viability, decrease cell apoptosis, and promote cell migration and angiogenesis under the intervention of glucocorticoids. Meanwhile, icariin intervention could reinforce these protective effects of BMECs-derived EVs. To sum up, the present study indicates that icariin acts as a promising candidate for treating glucocorticoid-induced injury of BMECs and bone diseases, partially through the autocrine activity of EVs. In vivo or animal studies are still required to better understand the function of BMECs-derived EVs.

## 1. Introduction

Synthetic glucocorticoids (GCs) (e.g., prednisolone and prednisone) are a class of anti-inflammatory and immunosuppressive agents widely prescribed in medical conditions, such as rheumatoid arthritis, bronchial asthma, severe acute respiratory syndrome (SARS), systemic lupus erythematosus, and acute urticaria [1]. Glucocorticoids bind to the glucocorticoid receptors (e.g., NR3C1, GR) on the cell surface and then target gene transcription in the nucleus, exerting an important role in physiologic function regulation [2]. Meanwhile, exogenous glucocorticoid administration is the principal iatrogenic cause for secondary osteoporosis and nontraumatic osteonecrosis of the femoral head (ONFH), which adds a tremendous economic burden to individuals and healthcare systems [1,2]. In patients with glucocorticoid-induced bone loss, angiogenesis is suppressed, accompanied by circulatory impairment, persistent bone destruction, increased apoptosis of osteocytes, disrupted balance of mesenchymal stem cells’ (MSCs) differentiation, fat embolism, and intramedullary pressure changes [3,4].

In addition to damaging bone tissues, glucocorticoids have shown a toxic effect on bone microvascular networks [5]. The bone microvascular endothelial cells (BMECs) constitute a monolayer structure attached to bony trabecula, contact with ingredients in the blood, and supply oxygen and nutrients to bone tissues [6]. Moreover, vascular endothelial cells possess secretory functions and can interact with bone cells by secreting multiple angiogenic and osteogenic factors (e.g., noggin, vascular endothelial growth factor (VEGF)), which is of great significance in the maintenance of bone mass [3,4]. Last but not least, bone microvasculature provides a specific microenvironment for the colonization of MSCs and other progenitor cells [6]. By elevating the production of hypoxia-induced factor-1α (HIF-1α) and reactive oxidative species (ROS), and downregulating the expression of endothelial nitric oxide synthase (eNOS), glucocorticoid could lead to injury and apoptosis of vascular endothelial cells, including BMECs [5]. Considering that normally functioning bone vessel networks are a precondition for skeletal development and regeneration, BMECs’ damage is believed to be a common mechanism for glucocorticoid-induced bone diseases [7]. There are multiple bioactive phytochemicals with protective effects on bone strength and vascular function, among which icariin was widely used to treat osteoporosis and ONFH [8].

Icariin (ICA/C33H40O15) (Figure S1, Supplementary Materials) is the main bioactive constituent of Epimedium brevicornum, a traditional Chinese herb known for “strengthening bone and tonifying kidney” for thousands of years [8,9]. Icariin could promote the proliferation and mineralization of osteoblasts and decrease the activity of osteoclasts [9]. Hu et al. revealed that icariin prevented injury and apoptosis of human umbilical venous endothelial cells following oxidized low-density lipoprotein treatment by regulating apoptosis regulators (caspase 3 and Bcl 2) [10]. Our prior studies demonstrated that intervention of icariin directly ameliorated glucocorticoid-induced injury of BMECs by altering the miRNA and protein expression profiles [11]. However, efforts to understand whether there were other underlying mechanisms are still ongoing.

The updated Guidelines of the International Society for Extracellular Vesicles (ISEV) on the minimal information for studies of extracellular vesicles (MISEV) provided recommendations on the experimental methods and minimal information required in the reporting and nomenclature of extracellular vesicles (EVs), a population of phospholipid bilayer-enclosed particles ranging from 20 nanometers to 10 microns or more in diameter [12]. The EVs could enable intercellular communication, immunoregulation, tissue regeneration, and the development of many pathologic conditions through transferring mRNAs, microRNAs (miRNAs), active proteins, and other biomolecules between cells, both at autocrine and paracrine levels, at short, medium, and long distances. Once stimulated, endothelial cells (ECs) will release EVs into the blood or extracellular fluid and the function of EVs originating from ECs are multi-faceted: on the one hand, they contribute to the onset of cardiovascular disorders (e.g., atherosclerosis) and, on the other hand, may enhance endothelial survival [13]. In recent years, it was confirmed that the autocrine activity of EVs could promote the development of multiple pathological changes such as diabetes mellitus type 2, glioblastoma, and ischemic diseases [14,15].

In this study, we aimed to investigate the role of the autocrine activity of ECs-derived EVs in the development of glucocorticoid-induced injury of BMECs and icariin intervention. Fractures associated with glucocorticoid-induced osteoporosis usually occur in regions with massive cancellous bone, especially the spine and proximal femur; meanwhile, osteonecrosis predominantly arises in the femoral head [16]. Given that ECs in different regions manifest morphological and functional specificity, BMECs isolated from the femoral head were used to conduct the current investigation.

## 2. Materials and Methods

This experimental protocol was designed following the World Medical Association Declaration of Helsinki on ethical principles for medical research involving human subjects and was approved by the Ethics Committee of China-Japan Friendship Hospital. The inclusion criteria were patients diagnosed as primary osteoarthritis (OA) of the hip, osteoarthritis secondary to developmental dysplasia of the hip (DDH), as well as femoral neck fracture, and subjected to total hip arthroplasty. Written informed consent must be signed before surgery. Eventually, BMEC isolated from a total of eighteen donors (mean age 59.9 ± 9.0 years, range: 43–77; F/M: 1/1; six primary OA, six OA secondary to DDH, and six femoral neck fractures) admitted to the orthopedic department of China-Japan Friendship Hospital from May 2017 to January 2021 were used to conduct the experiment presented herein.

### 2.1. BMECs Isolation

The BMECs were isolated following the methods described in the previous article [17]. The normal cancellous bone from the femoral head was processed into bone particles and washed with PBS thrice. These bone particles were digested with 2 mL 0.2% type I collagenase at 37 °C for 25 min, and then 3 mL 0.25% trypsin-EDTA for another 5 min. After filtration with a 70-µM cell filter, centrifugation was performed at 800 r/min for 5 min. The cell-debris pellet was re-suspended with 80 µL MACS running buffer and 20 µL CD31 microbeads. A total of 400 μL of MACS running buffer was added to the mixture, followed by slow passage over a magnetic field-immobilized MACS column. The cells on the sorting column were rinsed into a collection tube with 2 mL of DMEM buffer and incubated with endothelial cell culture medium (ECM catalog #1001, ScienCell, San Diego, CA, USA) in a cell incubator. Isolated cells were passaged when BMECs reached about 80% confluency. Purity of the isolated BMECs was evaluated through immunofluorescent staining. 

### 2.2. CCK-8 Assay

The BMECs were seeded in 96-well plates at 5 × 10^3^/well, cultured in a complete medium for 12 h, and treated with icariin (Solarbio Science and Technology Co. Ltd., Beijing, China; purity ≥98%) in different concentrations (0 M, 1 × 10^−6^ M, 1 × 10^−5^ M, 5 × 10^−5^ M and 1 × 10^−4^ M) for another 24 h. Icariin was dissolved in DMSO, whose final concentration in the organ bath solution did not exceed 0.1% and therefore would not cause a significant effect on the survival and proliferation of endothelial cells [18]. Then, BMECs were treated with hydrocortisone (0.3 mg/mL) (R&D Systems, Minneapolis, MN, USA) for another 24 h. A total of 10 μL of CCK8 reagent was added to 90 μL of culture medium per well and incubated for 4 h. Subsequently, the optical density (OD) at 450 nm was detected, using a microplate reader to calculate the cell proliferation.

### 2.3. EVs Isolation and Identification

After adherence, BMECs were cultured in an EVs-free medium (prepared by filtration through a 0.22 μm filter and then centrifugation at 100,000× *g* for 18 min) with and without icariin for 24 h. Then, the supernatant was collected to isolate EVs using the exosome isolation kit (MagCapture™ Exosome Isolation Kit PS, Wako, Japan). Each group of EVs was isolated from an equivalent number of BMECs and resuspended in PBS. The diameter distribution and volume of extracted EVs were analyzed using nanoparticle tracking analysis (NTA, Nanosight NS300, Malvern, UK) and the morphology was observed by transmission electron microscope (TEM; HT-7700, Hitachi, Tokyo, Japan). The EVs-carried proteins were assessed by Western blot assay using primary antibodies to CD9 (Santa Cruz Biotechnology, Santa Cruz, CA, USA; dilution 1:1000) and CD81 (Santa Cruz; dilution 1:800). 

### 2.4. High-Throughput Sequencing Transcriptome and Quantitative Polymerase Chain Reaction (qPCR) of EVs-Carried miRNAs

After extracting RNA from EVs by using TRIzol reagent, high-throughput sequencing of EVs-carried miRNAs was performed, according to standard procedures provided by Illumina. A small RNA sequencing library was prepared using the TruSeq Small RNA Sample Prep Kits (Illumina, San Diego, CA, USA). Then, the constructed library was sequenced using Illumina Hiseq 2000/2500 platform. MiRNAs with lower expression (those with less than 10 copies in all samples) were first removed from the analysis. Then, differentially expressed genes between BMECs in the icariin-treatment and the non-treatment groups were screened using |log_2_(Fold change)| > 1 and *p*-value ≤ 0.05 as the threshold. Then, genes related to proliferation, angiogenesis and apoptosis of endothelial cells were selected for confirmation by using qPCR. The reverse transcription process was performed using the One-Step PrimeScript^®^ miRNA cDNA Synthesis Kit (Takara, Shiga, Japan) and the amplification reaction was conducted using the SYBR^®^ Premix Ex TaqTM II kit (Takara, Japan). The primer sequences of investigated miRNAs were: hsa-miR-1469, forward, 5′-GCGCAGCTGGTAAAATGGAA-3′ and reverse, 5′-GTGCAGGGTCCGAGGT-3′; hsa-miR-133a-5p, forward, 5′-GCCTCGGCGCGGGGCGCG-3′ and reverse, 5′-GTGCAGGGTCCGAGGT-3′; hsa-miR-10b-5p, forward, 5′- GCTACCCTGTAGAACCGAA-3′ and reverse, 5′-GTGCAGGGTCCGAGGT-3′.

### 2.5. Bicinchoninic Acid (BCA) Analysis

Total proteins were extracted from EVs and measured using a BCA protein assay kit (Sigma, St. Louis, MO, USA). A total of 200 μL of BCA working solution was added to 10 µL of extracted protein sample per well of 96-well plates and incubated in the dark for 30 min at 37 °C. The OD value was measured at 750 nm, and the protein concentration was calculated from the standard curve, with bovine serum albumin as the standard protein.

### 2.6. Antibody Array Assay and Elisa Detection

The isolated proteins were scanned by using the GSH-CAA-440 array to obtain the raw data. Then, the Raybiotech software was used to remove the chip background and normalize the original data. Differentially expressed proteins (DEPs) between different groups were identified using the limma package (Version 3.42.2) in R software (RStudio, PBC, Boston, MA, USA). Individual *p*-values were calculated and converted to adjusted *p*-values (adj. *p*. val) for comparisons by false discovery rate correction of the Benjamini and Hochberg test. A cutoff point of adj. *p*. val < 0.05 and |fold change (FC)| > 1.2 were used to selected DEPs. Then, the PCA plot, heatmap, and volcano plot of the DEPs were drawn using the ggplot2 package in R software. The expression levels of specific protein of interest were determined by ELISA.

### 2.7. Functional Enrichment Analyses and Annotation of DEPs

The DEPs were uploaded to an online bioinformatics database, the Database for Annotation, Visualization, and Integrated Discovery (DAVID) version 6.8 Beta (https://david-d.ncifcrf.gov/ (accessed on 1st January 2022)), for enrichment analysis, including gene ontology (GO) and Kyoto Encyclopedia of Gene and Genome (KEGG) pathways. The obtained results were visualized in the R ggplot2 package and a *p*-value of <0.05 was considered statistically significant.

The protein–protein interaction (PPI) network was constructed by using the multiple protein online tool in the STRING database (version 11.0, http://string-db.org (accessed on 1st January 2022)) [19] and visualized in Cytoscape software (Version 3.6.2, Seattle, WA, USA). The targeted TFs and miRNAs of DEPs were predicted using the NetworkAnalyst platform (https://www.networkanalyst.ca/faces/home.xhtml (accessed on 1st January 2022)).

### 2.8. Coculture of BMECs and EVs

According to the intervention condition, BMECs were divided into three groups: in the Icariin-BMEC-EVs group, BMECs were cultured in the presence of EVs derived from BMECs treated with icariin; in the BMEC-EVs group, BMECs were co-cultured with EVs isolated from BMECs without icariin treatment, and in the Model group, BMECs were free from EVs’ intervention. In the former two groups, the EVs’ concentration was adjusted to 200 ng/uL. After 24 h of cell culture, all of the cells in the three groups were treated with hydrocortisone.

#### 2.8.1. Cell Scratch Assay

The BMECs were inoculated in 6-well plates and when the cells reached confluence as a monolayer, a 1 mL pipette tip was used to gently scratch the cell layer following the center lines of wells. An EVs and hydrocortisone (0.3 mg/mL) intervention was performed following the steps described, before and after 12 h and 24 h, images were taken under an inverted microscope.

#### 2.8.2. Tube Formation Assay

The BMECs, with and without EVs intervention, were inoculated to 24-well plates coated with enriched growth factor Matrigel (BD Biosciences, San Jose, CA, USA) for 24 h and then treated with hydrocortisone (0.06 mg/mL). After 4 and 8 h of hydrocortisone intervention, images were taken under the inverted microscope (Olympus, Tokyo, Japan).

#### 2.8.3. Flow Cytometry Assay

After 12 h of hydrocortisone (0.3 mg/mL) intervention, BMECs were harvested and washed twice with PBS at 4 °C and resuspended with 300 µL of binding buffer. A total of 100 μL of cell suspension, 5 μL of AnnexinV-FITC, and 10 μL of propidium iodide (PI) solution (20 ng/L) were mixed at room temperature, incubated in the dark for 15 min, and then the fluorescence intensity was detected by using a flow cytometer.

### 2.9. Statistical Analysis

Data are presented in the form of mean ± standard deviation (SD). The differences between groups were analyzed by the Student’s *t*-test and one-way analysis of variance (ANOVA). A *p*-value < 0.05 indicates a statistically significant difference. All statistical tests were performed using GraphPad Prism statistical software (version 6.0, San Diego, CA, USA).

## 3. Results

### 3.1. Cell Morphology Observation and Identification

**Figure 1:**
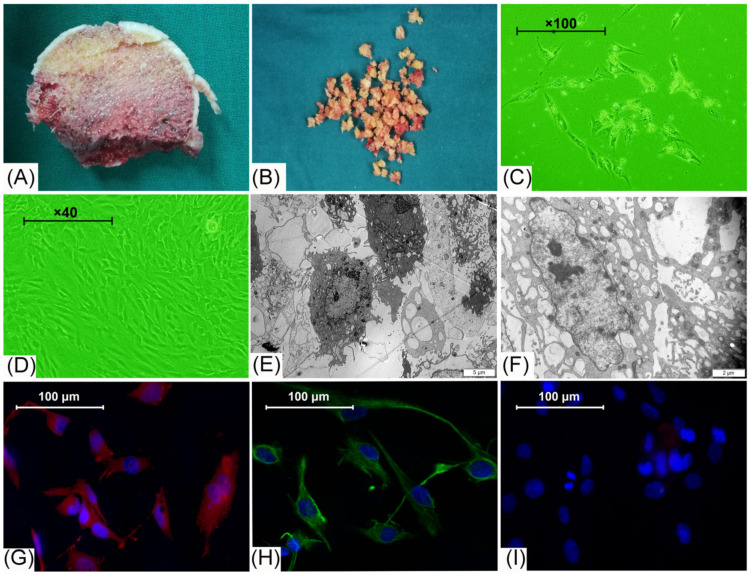
Obtaining of tissue sample, cell morphology observation, and purity identification. (**A**) Longitudinal section of the femoral head after femoral neck fracture; (**B**) bone particles for further isolation of cells; (**C**) three days after cell isolation, adherent cells evenly distributed in the bottom areas of the culture bottles, and were inconsistent in size and shape (100× magnification); (**D**) isolated cells show cobblestone morphology after 7 days of culture (40× magnification); (**E**,**F**) under scanning electron scope, cells were covered with microvilli and rich in organelles; (**G**–**I**) immunofluorescent staining shows high expression of CD31 (**G**) and vWF (**H**) and cells were negative for CD133 (**I**), red color indicates positive for CD31, green color indicates positive for vWF, and negative control images display cells stained with DAPI.

The normal cancellous bone from the resected femoral head (Figure 1A) was processed into bone particles (Figure 1B) to isolate the BEMCs. After three days of cell culture, it could be observed that adherent cells were evenly distributed in culture bottles (Figure 1C). When the isolated cells grew to 80% confluence, they became fusiform or polygonal and presented a cobblestone-like appearance under the inverted-phase contrast microscopy (Figure 1D). Under the transmission electron microscope, these cells were in a polygonal shape, with large ovular nuclei and prominent nucleoli. The cellular surface was decorated with microvilli formed by cytoplasm bulge; the cytoplasm was rich in mitochondria, ribosomes, rough endoplasmic reticulum, Golgi complexes, and endothelial-specific Weibel–Palade bodies (Figure 1E,F). Immunofluorescent staining of isolated cells revealed highly expressed vWF and CD31, but did not express CD133, indicating that these isolated cells were BMECs (Figure 1G–I).

### 3.2. Effects of Icariin on Cell Proliferation Rate

**Figure 2:**
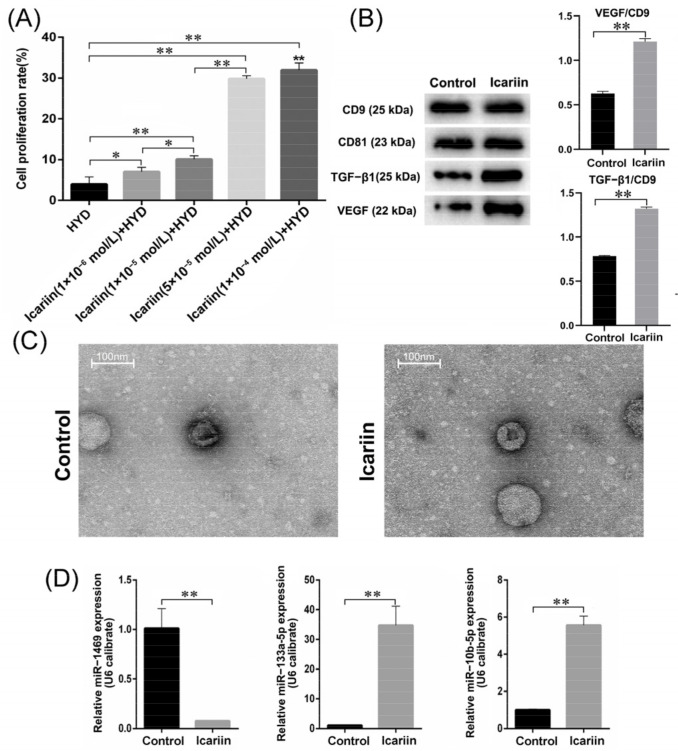
Icariin treatment and analysis of package in EVs. (**A**) Proliferation rate of BMECs increased with the concentration of icariin; effects of icariin in the 5 × 10^−5^ M and 1 × 10^−4^ M groups were most significant; (**B**) protein expression bands of VEGF, TGF-β1, CD9, and CD81 in the EVs isolated from BMECs in the icariin group and the control group; (**C**) morphology of isolated EVs from BMECs in the icariin group and the control group, as determined by transmission electron microscopy; (**D**) the expression level of hsa-miR-1469, hsa-miR-133a-5p, and hsa-miR-10b-5p in EVs isolated from the icariin group and the control group measured by using qPCR. HYD indicated hydrocortisone (0.3 mg/mL). Data analysis was performed using *t*-test analysis and ANOVA, error bars indicate standard deviation, and ** means *p* < 0.01, * means *p* < 0.05.

Compared with the icariin-free group, the proliferation rate of BMECs increased as the concentration of icariin increased (3.98% ± 1.76%, 7.04% ± 1.07%, 10.10% ± 0.83%, 29.86% ± 0.75% and 31.96% ± 1.75% in the 0 M, 1 × 10^−6^ M, 1 × 10^−5^ M, 5 × 10^−5^ M, and 1 × 10^−4^ M groups, respectively) (Figure 2A). There were significant differences among these groups (F = 207.5, *p*-value < 0.01). The proliferation promotion effects of icariin in the 5 × 10^−5^ M and 1 × 10^−4^ M groups were significantly better in comparison with the other groups (*p*-value < 0.01). Meanwhile, no significant difference was identified between these two groups (*p*-value = 0.18). So, 5 × 10^−5^ M was chosen as the optimal concentration for subsequent experiments.

### 3.3. Size and Protein Analysis of Isolated EVs

According to the NTA, for BMECs without icariin intervention (control group), the average diameter of isolated EVs was 86.68 ± 1.97 nm, while in the icariin group, the average diameter of EVs was 85.35 ± 1.65 nm. We found that icariin treatment did not significantly alter the diameter of EVs derived from BMEC cells in the two groups (*p* < 0.01). The control group had a relatively low concentration of EVs (9.85 ± 0.47 × 10^6^/mL). After being treated with icariin, the concentration of EVs increased to 1.54 ± 0.08 × 10^7^/mL. The BCA results showed that the protein content of conditioned media was 132.63 ± 3.33 μg/mL in the control group and 138.56 ± 9.58 μg/mL in the icariin group. The difference in protein content of conditioned media between the two groups was not statistically significant (*p* < 0.01). CD9 and CD81, two representative EVs surface markers, were highly expressed in EVs isolated from cells of both the control group and the icariin group (Figure 2B), indicating that isolated particles were EVs. Furthermore, the expression of VEGF and TGF-β1 protein was elevated in EVs derived from cells in the icariin group compared with those from the control group (*p* < 0.01) (Figure 2B). By TEM, the morphology of exosomes displayed classic spherical shape(Figure 2C).

### 3.4. MiRNA High-Throughput Sequencing and qPCR

A total of 50 differentially-expressed miRNAs between EVs isolated from BEMCs in the icariin and the control groups were identified, among which 20 miRNAs were downregulated and 30 miRNAs were upregulated after icariin treatment (Table S1, Supplementary Materials). EVs-derived miRNAs were reported to affect the angiogenesis of receipt cells. Two downregulated miRNAs (hsa-miR-133a-5p and hsa-miR-10b-5p) and one downregulated miRNA (hsa-miR-1469) were considered to potentially play important roles in the protective effect of icariin to glucocorticoid-induced injury of BMECs. The difference in the expression level of these three miRNAs was further confirmed by qPCR (*p* < 0.01) (Figure 2D).

### 3.5. Data Normalization and DEPs Identification

**Figure 3:**
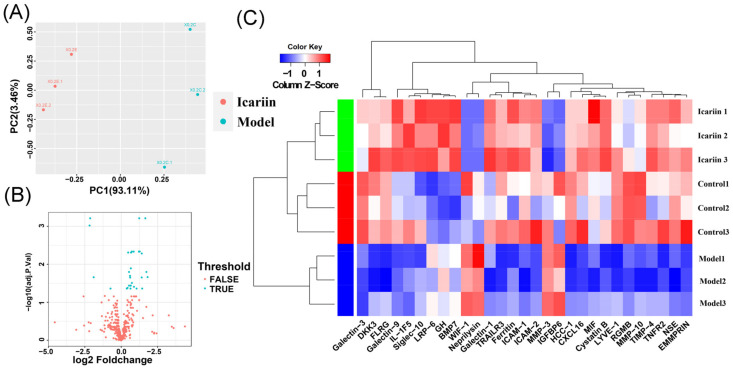
(**A**) Principal component analysis of different samples; (**B**) a volcano plot of identified protein in EVs isolated from BMECs in the Icarrin-treated group and the Model group. The blue dots represent DEPs (|FC| > 1.2 and adj. *p*. value < 0.05), the red dots represent proteins without significant difference. There were 25 differentially upregulated genes and 4 differentially downregulated genes; and (**C**) a heatmap of 29 differentially expressed proteins between the Icariin-treated group and the Model group. Hierarchical clustering analysis of z-scored FPKM was performed for each DEPs and the color scale represents FPKM normalized log10 transformed counts. Horizontal bars represent genes and the vertical column represents samples. Red color represents differentially upregulated genes, while blue color indicates differentially downregulated genes.

**Figure 4:**
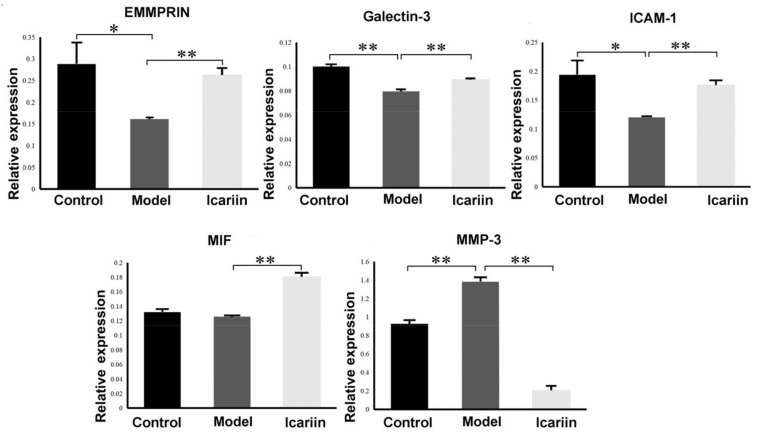
Relative expression level of five proteins identified by Elisa assay. Data analysis was performed using *t*-test analysis and ANOVA, error bars indicate standard deviation, and ** means *p* < 0.01, * means *p* < 0.05.

Then, the influence of icariin on cytokines in EVs secreted by BMECs were investigated with an injury model. Data normalization and cross-comparability were evaluated using the principal component analysis (PCA) to confirm biological variability between different EVs samples (Figure 3A). In total, 62 DEPs were identified between the EVs isolated from the Model group (BMECs treated with glucocorticoids) and the control group, including 11 upregulated and 51 downregulated DEPs listed in Table S2 (Supplementary Materials). Meanwhile, 29 DEPs were detected between EVs isolated from the Icariin-treated group and the Model groups, including 25 upregulated and 4 downregulated DEPs (Figure 3B). In addition, a heatmap were generated using the R ggplot2 package (Figure 3C). The clustering pattern showed that protein expression of EVs isolated from the Icariin-treated and Model groups was substantially different from each other. Elisa assay confirmed the difference in the expression level of five DEPs, including EMMPRIN, Galectin-3, ICAM-1, MIF and MMP-3 (*p* < 0.01) (Figure 4).

### 3.6. GO Function and KEGG Enrichment Analysis of DEPs

**Figure 5:**
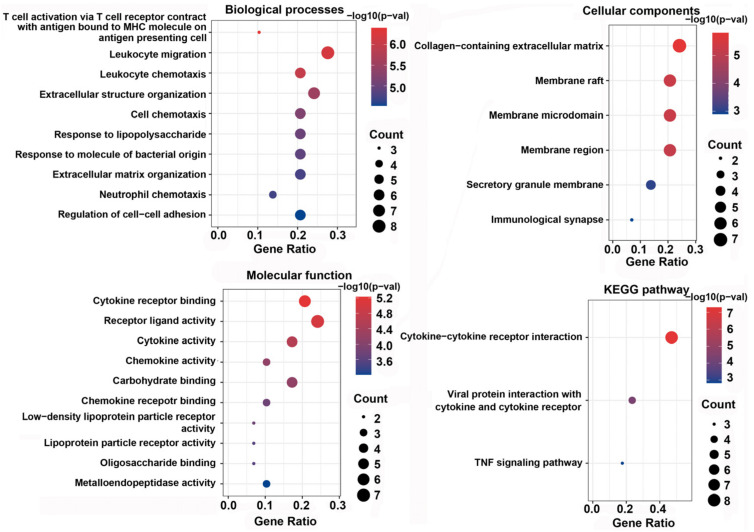
The most relevant biological processes (BPs), molecular functions (MFs), and cellular components (CCs) through GO enrichment analysis, and KEGG pathway enrichment analysis of differentially expressed proteins between EVs isolated from the Icariin-treated group and the Model group.

GO enrichment analysis of 29 DEPs between EVs isolated from the Icariin-treated group and the Model group was performed to identify the most relevant biological processes (BPs), molecular functions (MFs), and cellular components (CCs). The top ten enriched terms in BPs, CCs, and MFs are presented in Figure 5. Additionally, based on KEGG pathway analysis, the DEPs were significantly enriched in three signaling pathways, including cytokine–cytokine receptor interaction (eight proteins), viral protein interaction with cytokine and cytokine receptor (four proteins), and TNF signaling pathway (three proteins) (Figure 5).

### 3.7. PPI Network Construction and Hub Gene Selection

**Figure 6:**
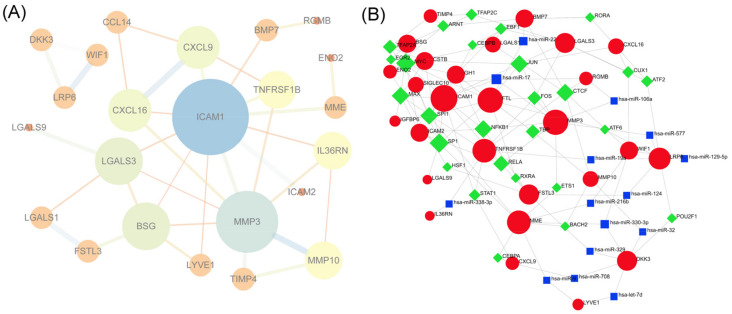
(**A**) The interactions between the 29 differentially expressed proteins between EVs isolated EVs from the Model group and the Icariin-treated group; (**B**) The TF–gene–miRNA interaction network.

The interactions between the 29 DEPs detected between EVs isolated from the Icariin-treated and the Model groups, which consisted of 24 nodes and 35 edges (Figure 6A), were constructed from the STRING database and visualized using Cytoscape. In addition, nine of the top genes with connectivity degrees ≥3 were: ICAM1 (Degree = 11), MMP3 (Degree = 8), BSG (Degree = 5), LGALS3 (Degree = 5), CXCL9 (Degree = 4), CXCL16 (Degree = 4), MMP10 (Degree = 3), IL36RN (Degree = 3), and TNFRSF1B (Degree = 3). The top nine hub genes were also selected by CytoHubba based on the MCC and MNC classification methods (Table 1), showing overlapping genes with different priorities.

### 3.8. TF-miRNA-DEP Interacted Network

The TF-miRNA interacted network was constructed through network analysis of 29 DEPs in Cytoscape (Figure 6B), which included 27 DEPs, 27 TFs, and 15 miRNAs, involving 99 associations between the TFs and DEPs, and 34 associations between the miRNAs and DEPs. We separately analyzed the degree of 27 DEPs in the TF-DEP network and the miRNA-DEP network (Table 2). Then, we found that MYC and SP1 regulated seven interacting DEPs. Simultaneously, hsa-miR-17 regulated five DEPs and hsa-miR-330-3p regulated three interacted DEPs.

### 3.9. Effect of BMEC-Derived EVs on BMEC Function

#### 3.9.1. Effect of EVs on Endothelial Cell Migration and Tube Formation

**Figure 7:**
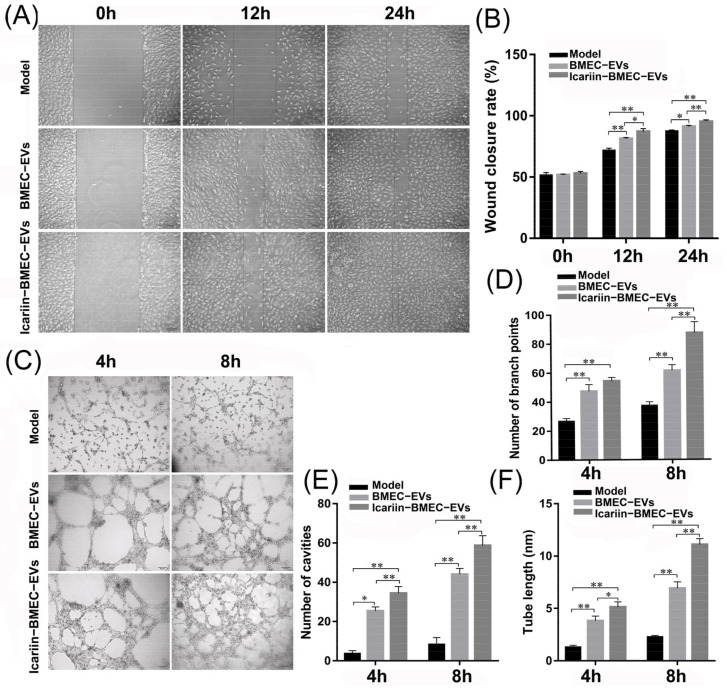
Effect of EVs on endothelial cell migration and angiogenesis. (**A**) Observation of scratch assay at 0, 12, and 24 h after hydrocortisone treatment; (**B**) scratch closure rate in three groups; (**C**) tube formation assay at 4 h and 8 h after hydrocortisone treatment; (**D**–**F**) number of branch points, number of cavities and tube length at 4 h and 8 h after hydrocortisone treatment. Data analysis was performed by *t*-test and ANOVA, with error bars indicating SD, ** indicating *p* < 0.01, and * indicating *p* < 0.05. Control indicates BMEC that were not co-cultured with EVs but treated with hydrocortisone; BMEC-EVs represent BMEC that were co-cultured with EVs secreted by BMECs without icariin intervention; and Icariin-BMEC-EVs indicate BMEC that were co-cultured with EVs secreted by BMECs treated with icariin and then treated with hydrocortisone.

After 12 h and 24 h of hydrocortisone treatment, the migration ability of BMECs pretreated with EVs was significantly higher than those without EVs treatment (*p* < 0.05) (Figure 7A,B). EVs pretreatment could also increase endothelial cells’ angiogenic capacity, including the number of branch points, the number of cavities and tube length (*p* < 0.05). According to the tube formation assay, the ability to promote migration and angiogenesis of microvascular endothelial cells was more pronounced in the Icariin-BMEC-EVs group in comparison with the BMEC-EVs group (*p* < 0.05), leading to a greater number of branch points, cavities, and longer tube length (Figure 7C–F).

#### 3.9.2. Cell Viability

**Figure 8:**
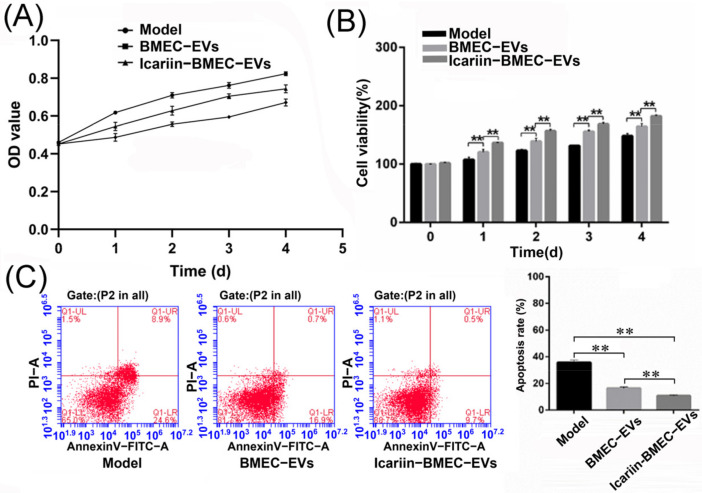
Co-culture with EVs improved the viability and decreased apoptosis of BMECs. These effects are more pronounced in Icariin-BMEC-EVs group than in BMEC-EVs group. (**A**) Detection of OD values at various time points after hydrocortisone intervention; (**B**) cell viability at each time point; (**C**) cell apoptosis detected by flow cytometry. Data were analyzed using one-way analysis of variance and student *t*-test, error bars indicate standard deviation, and ** indicates *p*-value < 0.01.

After treatment with hydrocortisone (0.3 mg/mL) for 0, 1, 2, 3, and 4 days, the viability of BMECs in the BMEC-EVs group and the Icariin-BMEC-EVs group was significantly enhanced in comparison with that of the Model group (*p*-value < 0.01). This effect was dominant in the Icariin-BMEC-EVs group compared with the BMEC-EVs group (*p* < 0.01) (Figure 8A,B).

#### 3.9.3. Cell Apoptosis Rate

After being treated with 1 mg/mL of hydrocortisone for 12 h, the apoptotic rates of BMECs in the BMEC-EVs group (16.47 ± 1.06%) and the Icariin-BMEC-EVs group (10.70 ± 0.7%) were lower than those in the Model group (35.67 ± 1.99%) (*p*-value < 0.01). Similarly, the inhibitory effect for apoptosis was more significant in the Icariin-BMEC-EVs group compared with the BMEC-EVs group (*p*-value < 0.01) (Figure 7C). A reduction of 70% in the apoptotic rate was observed in the Icariin-BMEC-EVs group in comparison with the Model group. These results indicated that the protective effect of icariin on the glucocorticoid-induced injury of BMECs was partially realized via the autocrine activity of BMECs-derived EVs.

## 4. Discussion

By using bone microvascular endothelial cells isolated from the femoral head, the experimental conditions are closer to the microvasculature in bone than those using human umbilical vein endothelial cells. The preceding finding has revealed that a hydrocortisone concentration of more than 0.3 mg/mL would induce typical proliferation inhibition and apoptosis of BMECs [11]. In this study, the isolated EVs exhibited classic morphological and biochemical features of EVs, and we demonstrated that autocrine activity of EVs ameliorated glucocorticoid-induced apoptosis of BMECs, as well as improved cell viability, migration, and angiogenesis. A great deal of the evidence investigated the clinical application of icariin, in which the dosage of icariin generally varied from 5 × 10^−6^ M to 5 × 10^−5^ M [20]. The present study has shown that exposure to icariin could remarkably enhance BMECs’ viability in a dose-dependent and dose-saturable manner and based on the results of CCK-8 assay, and 5 × 10^−5^ M was identified as the optimal concentration for promoting BMECs’ viability and reinforcing the protective effect of autocrine EVs for glucocorticoid-induced injury of BMECs. This effect was partially realized by changing the protein and RNA profiling in secreted EVs of bone microvascular endothelial cells.

The BMECs-derived EVs, defined as EVs released by BMECs during external activation, might be initiated by chemical (e.g., TNFα and IL-6) and physical (e.g., shear stress) factors [21]. The responses of endothelial EVs to various medications are demonstrably different. Zhao and his colleagues [22] showed that lipopolysaccharide increased the protein quantity of EVs released from pulmonary artery ECs, further enhancing proliferation and preventing apoptosis of pulmonary smooth muscle cells. TNF-α would not influence endothelial EVs concentration, but might alter EVs-carried protein and miRNA composition. For BMECs, icariin treatment did not significantly change the size and total protein level of secreted EVs. However, it could alter the composition of contents packaged in EVs, dramatically increasing the amounts of VEGF, TGF-β1, hsa-miR-133a-5p, and has-miR-10b-5p in EVs, while leading to lesser hsa-miR-1469. VEGF and TGF-β1 are often co-expressed in tissues and considered to be prominent promoters of angiogenesis; however, they show opposite effects for ECs [23]. The upregulated expression level of VEGF can be initiated by hypoxia, multiple cytokines, and growth factors, including TGF-β1. After combining with VEGF receptor 2 (VEGFR2), VEGF further transduces signals to downstream molecules (e.g., ERK1/2, c-Jun N-terminal kinase/JNK, phosphatidylinositol 3 kinase/PI3K, AKT, eNOS, and P70S6K), inhibits the apoptosis of ECs, and modulates a series of other physiological functions [24]. On the other hand, the possible mechanisms of TGF-β1 for enhancing angiogenesis and differentiation of ECs are manifold [25]. First, the optimal level of ECs apoptosis is a precondition for the formation of a functional vascular network, and inhibition of apoptosis may lead to abnormality of angiogenesis. Second, TGF-β1 could increase the expression of VEGF and other angiogenic factors in ECs. Third, ECs’ apoptosis induced by TGF-β1 is a transient process, occurring rapidly and followed by a prolonged refractory period. Last but not least, it was indicated that TGF-β1 promoted angiogenesis through the regulation of immune cells in vivo. Hsa-miR-133a-5p may protect against hypoxia/reperfusion-induced or ischemia/reperfusion-induced hepatocyte injury by decreasing the expression of MARK6 [26], while upregulation of hsa-miR-10b-5p could inhibit caspase-3 activation, and, therefore, prevent cell apoptosis [27]. Conversely, hsa-miR-1469 was demonstrated to induce apoptosis in a series of cancer cells [28].

ECs-derived EVs affects recipient cells’ function and modulates key events in the development of multiple disorders; the effect could be either beneficial or detrimental. It was also shown that capillary endothelial tip cells secrete EVs incorporating Delta-like 4 ligands, which are passed to the tip cells of the endothelial sprout, promote cell motility, and suppress their proliferation [29]. Senescent human ECs-derived EVs could knock down Frizled-3 of human MSCs and inhibit osteogenic differentiation [30]. Moreover, to systematically identify novel inflammatory cytokines related to the protective effect of icariin, we performed a screen among the Model group, Icariin-treated group and the control group by protein array assay. The present study found that 29 proteins were dysregulated in EVs secreted by BMECs after icariin intervention and most of these proteins were upregulated. Based on the PPI network, nine proteins (ICAM1, MMP3, BSG, LGALS3, CXCL9, CXCL16, MMP10, IL36RN, and TNFRSF1B) with high connectivity were identified as playing essential roles in the protective effect of icariin to glucocorticoid-induced injury of BMECs. ICAM1 is a cell surface glycoprotein typically expressed on endothelial cells [31]. Meanwhile, ICAM1 might exert dual effects on bone homeostasis, especially in osteoclastogenesis [32]. MMP3 and MMP10 belong to the matrix metalloproteinase family and also participate in osteogenic differentiation [33]. LGALS3, which is widely expressed in various tissues including bone tissues, is considered as a marker of bone formation [34]. The cytokine–cytokine receptor interaction pathway is predicted to be significantly enriched by identified DEPs, which included CCL14, TNFRSF10C, CXCL9, IL36RN, BMP7, TNFRSF1B, CXCL16, and GH1.

By constructing a TF-miRNA-DEP interacted network, we selected multiple important miRNAs (e.g., hsa-miR-330-3p and hsa-miR-17) and transcription factors (e.g., MYC and SP1) that might be involved in the dysregulation of these proteins. In former studies, these molecules were demonstrated to be vital for vascular development and angiogenesis [35,36]. MYC and SP1 could regulate the proliferation of endothelial cells after being stimulated by hyperoxia or hypoxia. Meanwhile, hsa-miR-17 is reported to affect the expression of these DEPs, and serves as a feasible strategy for the selective modulation of endothelialization and vascular remodeling [37]. It should be noted that these miRNAs and TFs were not differentially expressed in isolated EVs. In fact, they could interact with the identified DEPs in secretory cells or receipt cells, and therefore influence the cellular function.

This study is not without limitations. First, extrapolation of the results from cell models to clinical practice should be performed with caution. It is needed to consider the impact of factors in vivo. Second, although hsa-miR-133a-5p and hsa-miR-10b-5p were enriched after icariin intervention, we did not perform a further experiment to test whether they could take effect on the reversion of glucocorticoid-induced cellular injury. Last but not least, the interaction of EVs secreted by BMECs and other cell types needs to be further investigated. It is well-acknowledged that icariin could affect multiple cell types existing in bones, for example, MSCs and osteocytes; meanwhile, EVs can modulate inflammation, regulate the activation and migration of monocytes, and affect the differentiation of MSCs. Therefore, it is rational to assume that other kinds of cells in the bone or even cells of cardiovascular systems in the distance may also engulf BMECs-derived EVs may also be engulfed by different types of cells. In vivo and animal studies are required to confirm these findings and further illuminate the action mechanism of identified miRNAs and proteins for the protective effects of icariin to glucocorticoid-induced injury of the BMECs.

## 5. Conclusions

The present study indicates that the autocrine activity of extracellular vesicles could significantly improve glucocorticoid-induced injury of BMECs. Icariin intervention could reinforce these effects and may act as a promising candidate for glucocorticoid-induced bone diseases. In vivo or animal studies are still required to gain a better understanding of the function of BMEC-derived extracellular vesicles.

## Figures and Tables

**Table 1:** List of the top nine hub genes selected by MCC, MNC and degree methods in CytoHubba.

MCC	MNC	Degree
ICAM1	ICAM1	ICAM1
MMP3	MMP3	MMP3
BSG	BSG	BSG
LGALS3	CXCL9	LGALS3
CXCL9	CXCL16	CXCL9
CXCL16	LGALS3	CXCL16
MMP10	MMP10	MMP10
IL36RN	IL36RN	IL36RN
TNFRSF1B	TNFRSF1B	TNFRSF1B

MCC, maximal clique centrality; MNC, maximum neighborhood component.

**Table 2:** TF-miRNA-DEP interacted network.

MiRNAs	Targeted Genes	Gene Counts	Transcription Factors (TF)	Targeted Genes	Gene Counts
hsa-miR-17	ICAM1, MMP3, SIGLEC10, RGMB	4	MYC	ICAM1, BSG, ICAM2, FTL, LGALS1, CSTB, ENO2	7
hsa-miR-330-3p	DKK3, MME, WIF1	3	SP1	ICAM1, ICAM2, FTL, CSTB, SIGLEC10, LGALS9, MME	7
hsa-miR-22	FTL, LGALS1, BMP7	3	NFKB1	ICAM1, ICAM2, MMP3, GH1, FSTL3, TNFRSF1B	6
hsa-let-7d	DKK3, LYVE1	2	MAX	ICAM, BSG, ICAM2, IGFBP6, CSTB, TNFRSF1B	6
hsa-miR-106a	MMP3, RGMB	2	JUN	ICAM1, MMP3, FTL, CSTB, LGALS3, CXCL16	6
hsa-miR-124	LRP6, FSTL3	2	SPI1	ICAM1, FTL, IGFBP6, SIGLEC10, GH1, MME	6
hsa-miR-129-5p	LRP6, WIF1	2	CTCF	LGALS1, MMP10, BMP7, TNFRSF1B, WIF1, RGMB,	6
hsa-miR-19a	LRP6, TNFRSF1B	2	RELA	ICAM1, ICAM2, MMP3, FSTL3, TNFRSF1B	5
hsa-miR-216b	MME, WIF1	2	TFAP2A	BSG, CSTB, TIMP4, SIGLEC10	4
hsa-miR-32	DKK3, MMP10	2	FOS	MMP3, FTL, LGALS3, TNFRSF1B	4
hsa-miR-329	DKK3, FSTL3	2	TBP	MMP3, FTL, MMP10, GH1	4
hsa-miR-577	MMP3, LRP6	2	TFAP2C	BGS, LGALS3, TIMP4	3
hsa-miR-708	DKK3, CXCL9	2	STAT1	ICAM1, CXCL9, TNFRSF1B	3
hsa-miR-9	MME, LYVE1	2	EBF1	LGALS1, LGALS3, GH1	3

## Data Availability

Not applicable.

## Supplementary Materials

The following supporting information can be downloaded at: https://www.mdpi.com/article/10.3390/cells11121921/s1, Figure S1: Molecular formula of icariin; Table S1: Differentially expressed miRNA identified by high-throughput sequencing; Table S2: Differentially expressed proteins between the Model group and the Control group; Video S1: Video Abstract.
