# Supplementary material for: Autocrine Activity of Extracellular Vesicles Induced by Icariin and Its Effectiveness in Glucocorticoid-Induced Injury of Bone Microvascular Endothelial Cells
Source: Cells. 2022 Jun 14;11(12):1921. doi: 10.3390/cells11121921 (PMC9221798; doi:10.3390/cells11121921)
Supplement: Supplementary file 1 [file cells-11-01921-s001.zip › cells-1689845-supplementary.pdf]

Supplementary Table S1. Differentially expressed miRNA identified by high-throughput sequencing and associated literature review

Index	Name of microRNA	Icariin vs Control (up/down）	Fold change (Icariin/Control)	Expression level
1	hsa-miR-369-3p	down	0.27	middle
2	hsa-miR-101-5p	down	0.32	middle
3	hsa-miR-1469	down	0.36	middle
4	hsa-miR-135b-3p	down	0.37	middle
5	hsa-miR-505-5p	down	0.40	middle
6	hsa-miR-34a-3p	down	0.43	middle
7	hsa-miR-186-3p	down	0.47	middle
8	hsa-miR-212-5p	down	-infinite	low
9	hsa-miR-219b-5p	down	-infinite	low
10	hsa-miR-494-3p	down	-infinite	low
11	hsa-mir-1303-p5	down	-infinite	middle
12	hsa-mir-6516-p3	down	-infinite	low
13	hsa-miR-10527-5p	down	-infinite	middle
14	PC-3p-30597	down	-infinite	low
15	PC-5p-28622	down	-infinite	low
16	PC-3p-30534	down	-infinite	low
17	PC-5p-22551	down	-infinite	middle
18	PC-3p-33296	down	-infinite	low
19	PC-5p-28562	down	-infinite	low
20	PC-5p-27730	down	-infinite	low
21	hsa-miR-92b-5p	up	infinite	low
22	hsa-miR-101-2-5p	up	infinite	low
23	hsa-miR-214-3p	up	infinite	middle
24	hsa-miR-219b-3p	up	infinite	low
25	hsa-miR-335-5p	up	infinite	low
26	hsa-miR-374c-5p	up	infinite	middle
27	hsa-miR-760	up	infinite	low
28	hsa-miR-1249-5p	up	infinite	low
29	hsa-miR-3168	up	infinite	low
30	hsa-miR-4508	up	infinite	low

31	PC-3p-43313	up	infinite	low
32	PC-5p-42924	up	infinite	low
33	PC-5p-40409	up	infinite	low
34	PC-3p-42144	up	infinite	low
35	PC-5p-36973	up	infinite	low
36	PC-3p-43372	up	infinite	low
37	PC-3p-37275	up	infinite	low
38	PC-5p-37872	up	infinite	low
39	hsa-miR-363-3p	up	10.81	middle
40	hsa-miR-133a-3p	up	8.39	middle
41	hsa-miR-1-3p	up	8.02	middle
42	hsa-miR-133a-5p	up	6.62	middle
43	hsa-miR-133b	up	5.95	middle
44	hsa-miR-199a-3p	up	3.40	middle
45	hsa-miR-145-5p	up	3.02	middle
46	hsa-miR-219a-5p	up	2.93	middle
47	hsa-miR-199a-5p	up	2.56	middle
48	hsa-miR-10b-5p	up	2.30	middle
49	hsa-miR-338-5p	up	2.20	middle
50	hsa-miR-190a-5p	up	2	middle

Supplementary Table S2. Differentially expressed proteins between the Model group and the Control group.

Protein ID	Fold change	Adj.p.val	Regulation
S100AB	56.22641801	0.013139834	up
CD40	3.871571268	0.007662016	up
MME	2.72198199	0.00825969	up
ROBO3	2.670461172	0.000640298	up
SHH	2.32282831	0.005645997	up
IL32	2.273986519	0.01810351	up
VEGFA	2.062416237	0.004967094	up
NRCAM	1.555622839	0.016332655	up
FGF4	1.522294946	0.02028389	up
BMPR1A	1.369496546	0.027545274	up
GH1	1.300379745	0.03037901	up
LGMN	0.81409151	0.022241753	down
AFP	0.802656053	0.036886707	down
DKK3	0.715824206	0.0338396	down
IL36G	0.706879901	0.02028389	down
FSTL3	0.68470189	0.008618897	down
SCARB2	0.670054713	0.043089497	down
LGALS9	0.668097473	0.022241753	down
FSTL1	0.66750771	0.028256696	down
CXCL1	0.667026409	0.0338396	down
FLT1	0.666415012	0.022241753	down
CDH3	0.655557701	0.043089497	down
CLU	0.644129443	0.045346808	down
IL6R	0.635128283	0.014263345	down
TNFRSF11B	0.634876117	0.010530573	down
TNFRSF11A	0.626375674	0.025195159	down
CRTAM	0.623938841	0.0338396	down
TNFRSF1A	0.606342841	0.036886707	down
PLAUR	0.603419501	0.009866887	down
LDLR	0.583130282	0.008618897	down
LTA	0.580573864	0.036886707	down
TIMP4	0.565981807	0.049107913	down
FAS	0.56021499	0.010080287	down
CXCL16	0.560155743	0.029013949	down
IGFBP1	0.554532181	0.0338396	down
BSG	0.551867994	0.008834354	down
PI3	0.548277818	0.030895065	down
DSG2	0.512840553	0.019254657	down
AXL	0.510493425	0.009579491	down
CDH13	0.473281965	0.006680472	down
CCL26	0.46809368	0.039275963	down
TNFRSF1B	0.462345269	0.03749305	down
TNFRSF10C	0.460391199	0.005349222	down

REN	0.440865129	0.010530573	down
LGALS3	0.434429603	0.000197865	down
ICAM1	0.417325988	0.047383236	down
RBP4	0.41431661	0.022241753	down
MMP10	0.41247727	0.000474226	down
CCL23	0.411568467	0.00020881	down
ENO2	0.401547029	0.001485212	down
BDNF	0.390946018	0.0338396	down
IGFBP4	0.382464021	0.046300423	down
LIF	0.37277882	0.001485212	down
IL6R	0.370503352	0.047581298	down
RGMB	0.333312098	3.78E-05	down
DLL1	0.299374369	0.0338396	down
TFPI	0.292241474	0.000424348	down
IL11	0.250549019	0.000409466	down
CCL14	0.221447435	3.78E-05	down
LYVE1	0.186006543	0.001200941	down
CCL15	0.12458644	0.000414012	down
CCL8	0.100239176	0.013439777	down

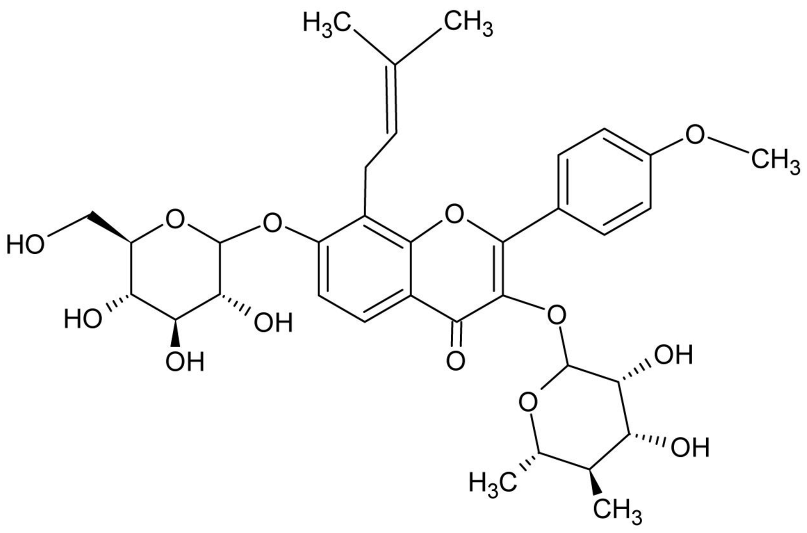

Supplementary Figure S1. Molecular formula of icariin.
